# COVID-19 Protective Behaviors Are Forms of Prosocial and Unselfish Behaviors

**DOI:** 10.3389/fpsyg.2021.647710

**Published:** 2021-04-09

**Authors:** Bojana M. Dinić, Bojana Bodroža

**Affiliations:** Department of Psychology, Faculty of Philosophy, University of Novi Sad, Novi Sad, Serbia

**Keywords:** COVID-19, coronavirus, protective behaviors, selfishness, prosociality, empathy, fear

## Abstract

The aim of this study was to explore the effects of prosocial and antisocial personality tendencies and context-related state factors on compliance with protective behaviors to prevent the spread of coronavirus infections. Six types of prosocial tendencies (altruism, dire, compliant, emotional, public, and anonymous) and selfishness as the antisocial tendency were included as personality factors, while fear related to the pandemic and empathy toward vulnerable groups (i.e., those in forced isolation) were context-related factors. Furthermore, mediation effect of empathy and moderation effect of fear were explored in relations between personality factors and protective behaviors. The sample included 581 participants (78.3% females). The data were collected from March 28 to April 6, 2020, during the emergency state and curfew in Serbia. The results showed that tendency to help anonymously had a positive effect and selfishness had a negative effect on protective behaviors, over and above demographic characteristics and context-related factors. Among context-related factors, only fear related to the pandemic had a significant unique positive effect on protective behaviors, but it had no moderator effect in the relationship between personality traits and protective behaviors. However, empathy acted as a mediator and partly accounted for the negative effect of selfishness and positive effect of tendency to help anonymously on protective behaviors. The results revealed that compliance with protective measures could be seen as prosocial and unselfish form of behavior. Furthermore, these findings have practical implications for shaping public messages and they can help effectively promote health-responsible behaviors.

## Introduction

In order to contain the spread of COVID-19 infections, the World Health Organization proclaimed several protective measures, such as physical distancing, wearing a mask, avoiding crowds, and cleaning hands (World Health Organization, 2020). These protective behaviors that serve to keep a person safe and protected from the virus infection could also be seen as prosocial behaviors. For example, by wearing a mask and keeping a physical distance, people protect others, especially those most vulnerable to the virus, such as the elderly and individuals with respiratory problems. However, in a review of evolutionary insights into the understanding of the pandemic's impact on human behavior, Seitz et al. (2020) stated that it was unclear whether protective behaviors referred to cooperative motives and solidarity among people or to concerns about oneself and close family members and worries about social shaming and legal sanctions.

From the standpoint of evolutionary psychology, the main goal of individual behaviors is the pursuit of survival and reproduction (Buss, 2019). Our behaviors are consequences of successful problem-solving strategies of our ancestors who passed down these adaptive behaviors to subsequent generations. However, strategies for securing resources can vary. There are two kinds of strategies that have evolved, prosocial and antisocial (e.g., Gilbert and Basran, 2019). The prosocial strategy can help in providing resources and securing reproductive opportunities by ensuring mutual advantages in terms of breeding, offspring care, and a cooperative alliance. The antisocial strategy includes competition for resources, both within and between groups, in an environment in which only the strongest wins. Thus, in the global crisis of the COVID-19 pandemic, the question is which strategy underlies protective behavior. On the one hand, there is the long-held popular view that human nature is inherently self-serving and selfish. However, challenging contexts, such as the COVID-19 pandemic, may actually promote altruism (Vieira et al., 2020). Prosocial behaviors could be driven by altruistic motives focused on maximizing others wins or egoistic motives focused on maximizing own wins. In this study six types of prosocial tendencies were explored and they ranged from self-oriented (i.e., public prosociality as a tendency to perform prosocial acts in front of an audience, motivated by the desire to gain the approval of others) to other-oriented (i.e., altruistic and anonymous prosociality as a tendency to perform prosocial acts without knowledge of whom helped, see Carlo and Randall, 2002).

Previous research has yielded mixed results about the prosocial correlates of compliance with protective behaviors. For example, Pfattheicher et al. (2020) showed that empathy was related to physical distancing and wearing a mask, while inducing empathy for people most vulnerable to the virus promoted the motivation to adhere to protective behaviors. Greater empathy toward vulnerable others along with greater perception of social cohesion significantly predicted support for protective measures (Böhm et al., 2020). Furthermore, the motivation to engage in protective behaviors was increased by public health appeals more than by personal health appeals (Jordan et al., 2020).

However, other authors reported no significant relation between altruism and some protective behaviors, such as social distancing (Sheth and Wright, 2020). Moreover, Nakayachi et al. (2020) investigated the reasons for wearing a mask among the Japanese and found that the perceived self-efficacy of wearing a mask in reducing personal infection risk had a higher correlation with mask usage compared to the reduction of risk for others. However, both reasons still had negligible effects in the prediction of mask usage (Nakayachi et al., 2020). The most prominent predictor for wearing masks was conformity to the social norms, followed by the feeling of relief from anxiety (Nakayachi et al., 2020). Compliance with protective measures has been one of the main themes in papers related to fear and anxiety triggered by COVID-19 (Coelho et al., 2020). In the context of prosocial behaviors, fear, anxiety, and personal distress force individuals to focus on their own emotions and personal losses as opposed to others' gains (Paciello et al., 2013). Consequently, they motivate people to act in a self-interested manner, i.e., to ensure personal survival. Many studies have confirmed the link between personal fear of COVID-19 and protective behaviors (e.g., Harper et al., 2020). However, in some studies, the measure of worry about the consequences of the novel coronavirus was not separated from worry for oneself, family, and close friends, but the overall score on worry showed a substantive positive correlation with protective behaviors across different countries (Jørgensen et al., 2020). Therefore, fear of the pandemic should be taken into account as a strong context-related state factor in the explanation of compliance with protective measures.

In answering the question of whether prosocial or antisocial tendencies could explain compliance with protective measures, previous studies that included basic personality traits also showed mixed results. Among the Big Five traits, the trait related to prosociality, empathy, and helping behavior is Agreeableness (Graziano et al., 2007). While the majority of studies reported positive relations between Agreeableness and protective behaviors during the pandemic (Aschwanden et al., 2020; Blagov, 2020; Bogg and Milad, 2020), some studies did not find significant relations (Shook et al., 2020), or they found even negative relations (Abdelrahman, 2020). In the context of the HEXACO personality model, Honesty-Humility reflects active cooperation, the tendency to cooperate with others despite the opportunity for exploitation, while Agreeableness reflects reactive cooperation, the tendency to cooperate with others despite their misgivings (Ashton et al., 2014). However, meta-analysis showed that only Honesty-Humility was related to prosociality and not Agreeableness from the HEXACO model (Thielmann et al., 2020). In the same vein, previous research has found Honesty-Humility, but not Agreeableness, to be positively related to support for limited social gatherings and the closing of restaurants (Böhm et al., 2020). Similar, (Zettler et al., 2020) showed significant effect of Honesty-Humility on distancing, while Agreeableness had no significant effect on both distancing or hygiene, although both traits showed significant correlations with those protective behaviors. Conversely, the constellation of socially aversive traits known as the Dark Triad has been consistently linked to non-compliance with protective measures (e.g., Nowak et al., 2020; Triberti et al., 2021; Zettler et al., 2020). Dark Triad traits refer to antisocial strategies that share common characteristic of manipulativeness and lack of affective responsivity or empathy (Dinić et al., 2020). Furthermore, one of the core elements of this constellation could be selfishness (Diebels et al., 2018). Additionally, Moshagen et al. (2018, p. 656) defined the common core of dark traits or D factor as “the tendency to maximize one's individual utility-disregarding, accepting, or malevolently provoking disutility for others-, accompanied by beliefs that serve as justifications” which also refers to selfishness. However, prosocial and antisocial tendencies are not merely opposite sides of the same dimension. Rather, they form related but distinct constructs (e.g., Krueger et al., 2001). Thus, it seems important to include both prosocial and antisocial tendencies as predictors in explorations of the nature of protective behaviors.

The main aim of this research was to explore the effects of specific personality traits and tendencies related to prosociality (prosocial tendencies) and antisociality (selfishness) along with the effects of context-related factors (fear related to the pandemic and empathy toward people in forced isolation) on compliance with protective measures. Previous research suggested that context-related factors were more important and outperform the personality traits in explanation of protective behavior (e.g., Zajenkowski et al., 2020). However, other research suggested the important role of personality traits (Zettler et al., 2020). Additionally, previous research suggested that demographic characteristics need to be considered in exploration of protective behaviors (e.g., Lüdecke and von dem Knesebeck, 2020). Therefore, in order to gain insight into the main characteristics of protective behaviors and determine whether they reflect prosocial or selfish tendencies, compared to previous research (e.g., Blagov, 2020), we explored the effects of personality traits in the explanation of compliance with protective measures over and above context-related factors and demographics. In this way we controlled the effects of context-related factors and demographics in relations between prosocial and antisocial tendencies and protective behaviors. We expected that the practice of protective behaviors would be positively affected by other-oriented prosocial tendencies (such as altruism) and negatively affected by selfishness and self-oriented prosocial tendencies (such as public prosocial tendency).

The second aim was to explore the mediation and moderation role of context-related factors in relations between personality traits and protective behaviors. Most studies have confirmed the strong relation between protective behaviors and empathy, especially toward the vulnerable ones (e.g., Pfattheicher et al., 2020). Since antisocial or “dark” tendencies are negatively linked to empathy (e.g., Dinić et al., 2020), we expected selfishness and self-oriented prosocial tendencies to be negatively related to protective behaviors due to a lack of empathy, i.e., that empathy acts as a mediator. Conversely, we expected that positive effects of other-oriented prosocial tendencies on protective behaviors could be explained by higher empathy. In the case of fear, we assumed that it could act as a moderator. Namely, fear of COVID-19 appears to be a strong correlate of protective behaviors (e.g., Coelho et al., 2020). However, there are no theoretical arguments for relations between prosocial and antisocial tendencies and fear related to pandemic. Although we expected that selfishness and self-oriented prosocial tendencies decrease protective behavior, if the fear is high among those with higher selfishness and self-oriented prosocial tendencies, we assumed that it would lead to higher compliance with protective measures. In the same vein, we expected that a positive link between other-oriented prosocial tendencies and protective behaviors would increase in the case of higher fear.

## Method

### Participants and Procedure

The sample included 581 participants (78.3% females) from Serbia, aged between 19 and 72 (*M* = 34.01, *SD* = 10.27). The majority of participants were highly educated (50.8% university graduates, 10.3% university postgraduates or PhD students, 21.7% students, and 6.0% finished college), while 11.2% finished primary or secondary school. Participants reported 1 (meaning they lived alone) to 12 household members. Due to the small frequencies of participants in households with more than 6 members, these answers were merged into one category (*M* = 3.06, *SD* = 1.34).

Participants were invited to take part in the study through a social media announcement. The data were collected from March 28 to April 6, 2020 (the 2nd and the 3rd week of the emergency state in Serbia). The study was a part of a larger research project, which was approved by the Ethical Committee of the Department of Psychology, Faculty of Philosophy, University of Novi Sad, Serbia, which is the Second Instance Commission of the Ethical Committee of the Serbian Psychological Society (No. 202003221959_nytc). A part of the data was also used in Dinić and Bodroža (2020).

### Instruments

The COVID-19 Protective Behaviors Scale was developed for the purpose of this study. It contains 9 items (e.g., washing hands, wearing a mask, wearing sanitary gloves, and physical distancing) with a 5-point Likert scale for the frequency of each behavior (from 0 = never to 4 = all the time). Based on the principal axis method, only one factor had an eigenvalue over 1 (λ = 3.08), which explained 24.26% of the common variance. Factor loadings ranged from 0.36 (“consciously prevent yourself from touching your face with your hands when you are outside”) to 0.74 (“disinfect your shoes when you get home”). The mean score was 3.63 (*SD* = 0.87) and the alpha was 0.82.

The Empathy Toward Persons in Forced Isolation Scale was developed for the purpose of this study. It contains 6 items (e.g., “I get very sad when I think of people who are forced into total isolation.”) with a 5-point scale (from 1 = never to 5 = always). Based on the principal axis method, only one factor had an eigenvalue over 1 (λ = 2.44), which explained 40.66% of the common variance. Factor loadings ranged from −0.41 (“Talks and messages about helping people in isolation irritate me.”) to 0.77 (“I am thinking about people who are in forced isolation and the situation in which they are.”).

The Fear scale from the Positive and Negative Affect Schedule (PANAS; Watson et al., 1988, for the Serbian adaptation see Mihić et al., 2014) contains 5 items. Participants were asked to judge on a 5-point scale (from 1 = *not at all* to 5 = *very much*) how they felt since the COVID-19 pandemic started in Serbia.

The Selfishness Questionnaire (SQ; Raine and Uh, 2019, for the model fit of the Serbian adaptation see Dinić and Bodroža, 2020) contains 24 items with a 5-point scale (from 1 = *strongly disagree* to 5 = *strongly agree*) that measure adaptive (selfish acts with benefits for oneself and close persons such as family and friends), egocentric (a single-minded attentional focus on the self), and pathological selfishness (inflicting harm upon others for self-advancement purposes).

The Prosocial Tendencies Measure (PTM; Carlo and Randall, 2002, for the model fit of the Serbian adaptation see Dinić and Bodroža, 2020) contains 23 items on a 5-point scale (from 1 = *does not describe me at all* and 5 = *describes me greatly*) that measure six types of prosocial tendencies: altruism (voluntary helping motivated primarily by one's concern for the needs and welfare of others), compliant (helping others in response to a verbal or non-verbal request), emotional (helping others under emotionally evocative circumstances), dire (helping in crises or emergencies), public (helping in front of an audience, at least partially motivated by the desire to gain the approval and respect of others and enhance one's self-esteem), and anonymous (helping without others being aware of who helped them). Among them, public could be seen as purely egoistic, self-oriented prosocial tendency, altruism as a purely other-oriented tendency, while the rest of them could be sorted out between these extreme categories.

Descriptives and alpha reliabilities are presented in Table 1. Reliabilities are consistent to those obtained in the previous studies, including somewhat lower reliability for altruism and dire prosocial tendency, which are low, but still acceptable considering small number of items (e.g., Carlo and Randall, 2002; Carlo et al., 2003; Raine and Uh, 2019). Data and instruments could be found at https://osf.io/gdz42/.

**Table 1:** Contributions of demographics, context-related, and personality factors to COVID-19 protective behaviors and correlations.

**Variables**	**β**	***r***	***M***	***SD***	**α**
**Control variables:** ***R***^**2**^ **=** 0.09^***^					
Sex	0.15^***^	0.16^***^ (*r*_bs_)	–	–	–
Age	0.16^***^	0.16^***^	34.01	10.27	–
Household size	0.09^*^	0.09^*^ (ρ)	3.06	1.34	–
**Context-related factors:** **Δ*****R***^**2**^ **=** 0.04^***^
Empathy toward people in forced isolation	0.03	0.20^***^	3.91	0.72	0.78
Fear related to the pandemic	0.19^***^	0.19^***^	2.78	1.01	0.90
**Personality factors:** **Δ*****R***^**2**^ **=** 0.04^***^					
Selfishness	−0.13^**^	−0.21^***^	2.04	0.60	0.90
Dire	0.04	0.09^*^	3.75	0.78	0.54
Public	0.02	−0.02	1.43	0.60	0.78
Anonymous	0.10^*^	0.20^***^	3.29	0.97	0.81
Compliant	0.03	0.15^***^	4.12	0.80	0.78
Emotional	0.02	0.11^**^	3.73	0.86	0.77
Altruism	0.02	0.08^*^	4.29	0.58	0.55
Total *R*^2^ = 0.17^***^					

Sex was coded as 1 = male and 2 = female; ρ, rho rang correlation; r_bs_, point-biserial correlation;

*** *p < 0.001*,

** *p < 0.01*,

* *p < 0.05*.

## Results

### Effects of Demographics on Protective Behaviors

The results showed that compliance with COVID-19 protective behaviors was more frequent among women [*t*_(577)_ = −5.25, *p* < 0.001, *M*_female_ = 3.73, *SD*_female_ = 0.84, *M*_male_ = 3.23, *SD*_male_ = 0.89], older people, and those who live in households with more people (Table 1), but the correlation with educational level was not significant (ρ = 0.07, *p* = 0.103). Thus, sex, age, and household size would be added as covariates in further analyses in order to control their effects on explored relations.

### Correlations Between Protective Behaviors and Context-Related and Personality Factors

Considering context-related factors, both empathy toward people in forced isolation and fear related to pandemic were positively related to protective behaviors (Table 1). Considering personality factors, all prosocial tendencies were positively related to protective behaviors, except for the tendency toward public prosocial behavior, which showed no significant correlation. Mutual correlations between three selfishness subscales were high (0.64, 0.65, and 0.73) and all three scales showed relatively similar intensity of negative relations with protective behaviors (from −0.17 to −0.19, all *p*s < 0.001). Thus, the total score of selfishness was used in further analyses and it showed low negative correlation with protective behaviors (the remaining correlations are reported in Supplementary Table A). Therefore, both context-related and individual factors showed significant correlations with protective behaviors. However, it should be noted that all correlations were small.

### Prediction of Protective Behaviors

To explore the prediction of protective behaviors based on demographic, context-related, and personality factors, a hierarchical regression analysis was conducted. In the first step, sex, age, and household size were entered to control their effects. In the second step, context related factors (empathy toward people in forced isolation and fear related to the pandemic) were entered. In the third and final step, personality factors (selfishness and prosocial tendencies) were entered. The results showed that both context-related and personality factors significantly contributed to the prediction of compliance with protective behaviors. Personality factors had a significant contribution over and above demographic characteristics and context-related factors (Table 1). Among context-related factors, only fear related to the pandemic had a significant positive contribution to compliance with protective behaviors. Although empathy was significant in the second step (β = 0.12, *p* < 0.01), with the inclusion of personality traits, it became a non-significant predictor. Among personality factors, only selfishness and anonymous prosocial tendency had significant contributions, in opposite directions.

### Mediation Effect of Context-Related Factor of Empathy

**Figure 1:**
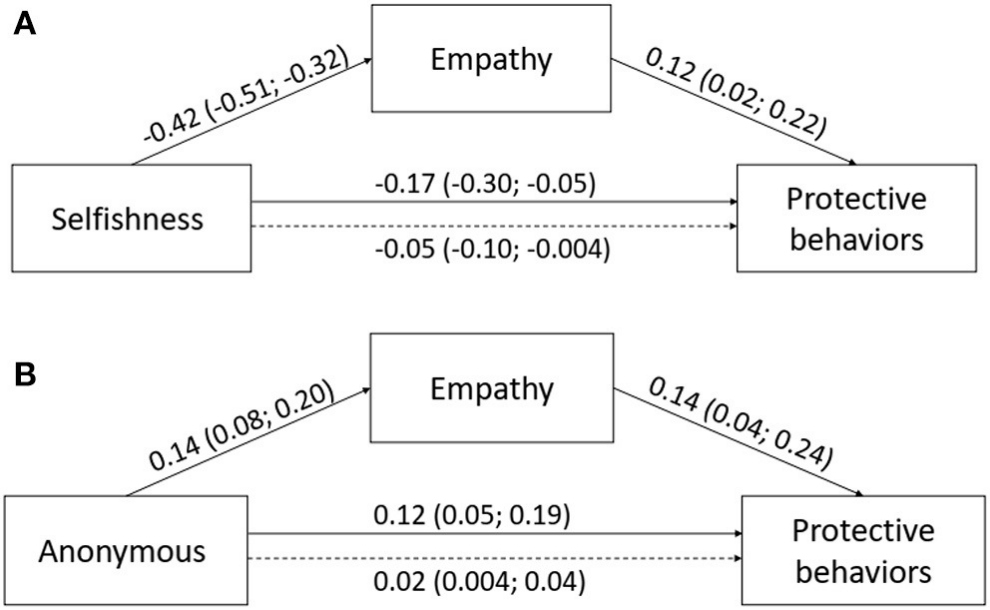
Mediation effect of empathy in relations between selfishness **(A)** and anonymous prosociality **(B)** with protective behaviors. Unstandardized beta coefficients and 95% CIs were presented. Betas below dotted line refers to indirect effects.

Mediation effects of empathy toward persons in forced isolation in relations between personality factors and protective behaviors were tested, with sex, age, and household size as covariates in order to control their effects (analyses were conducted in PROCESS macro for SPSS v.3.4, Hayes and Little, 2018). In the mediation analysis, only traits that had a significant contribution to protective behaviors were tested, i.e., selfishness and anonymous prosocial tendency. In the case of selfishness as a predictor, the mediation effect of empathy was significant (Figure 1A). Empathy acted as a buffer and weakened the negative effect of selfishness on compliance with protective measures. In the case of anonymous prosociality as a predictor, empathy was also significant mediator and partly explained of the effect of anonymous prosociality on protective behaviors (Figure 1B).

## Moderation Effect of Context-Related Factor of Fear

Moderation analysis showed no significant interaction effect of fear related to pandemic and selfishness (Δ*R*^2^ = 0.003, *p* = 0.14) on protective behaviors, with control of demographic variables. Additionally, there is no significant interaction between fear and anonymous prosocial tendencies (Δ*R*^2^ = 0.001, *p* = 0.36) on protective behaviors.

## Discussion

The main result of this study is that both selfishness and prosocial tendencies had effects on protective behaviors over and above demographic and context-related factors, but in opposite directions. Thus, selfishness had negative effects on compliance with protective measures, meaning that more selfish people are less likely to adhere to health-protective measures. Among prosocial tendencies, all but public prosocial tendency showed a significant positive correlation with protective behaviors. However, in the regression analysis, only anonymous prosocial tendency showed a unique significant contribution. Anonymous prosocial tendency is defined as helping without knowledge of who helped (Carlo and Randall, 2002). In previous studies, it showed no significant correlation with altruism or with Big Five personality traits (e.g., Rodrigues et al., 2017). However, it was positively related to the aspects of both cognitive and affective empathy and global prosocial behavior, while it was negatively related to hedonistic prosocial moral reasoning (Carlo and Randall, 2002; Carlo et al., 2003; Rodrigues et al., 2017). It could be assumed that those who are more other-oriented and prone to anonymous prosocial behavior are less concerned with personal desires and needs and they are characterized by higher emphatic concern, which leads them to practice protective measures. Indeed, a further analysis showed that empathy acts as a mediator in the positive relation between anonymous prosociality and protective behaviors. Additionally, health-irresponsible behaviors among more selfish people could be partially explained by the lack of empathy. This is important from the standpoint of formulating public communication to promote positive behavior change, which should be referred to as protection of the most vulnerable groups and finally, of all others, compared to the protection of oneself (see Jordan et al., 2020).

The results are generally in line with the expectation that prosocial tendencies would be positively related and that antisocial tendencies would be negatively related to compliance with protective measures. However, the non-significant contribution of altruism was not expected. Altruism showed a small positive correlation with protective behaviors, but it was not among the significant unique predictors of these behaviors. Altruism in the PTM refers to helping others when there is little or no perceived potential for a direct, explicit reward to the self (Carlo and Randall, 2002). It is negatively related to approval-oriented prosocial moral reasoning and personal distress and positively related to Agreeableness, but it is unrelated to indicators of empathy and global prosocial behavior (Carlo et al., 2003; Rodrigues et al., 2017). Thus, it seems that global empathic capacity could make the distinction between altruism and anonymous prosocial tendencies. The results support the view according to which the tendency that is linked to empathy obtains the main effect on the practice of protective behaviors (e.g., Pfattheicher et al., 2020). Additionally, in this study, empathy showed a higher correlation with anonymous prosociality (*r* = 0.22) than with altruism (*r* = 0.11, *Z* = 1.97, *p* = 0.048).

In the same vein, one could expect that emotional prosociality had significant effect in predction of protective behaviors. However, emotional prosociality refers to help under emotionally evocative circumstances, e.g., in the presence of obvious physical pain or distress. Since pandemic do not include such demands as situations that call for fast reacting (as, for example, presence of physical pain), we could assume that this is the reason why emotional component was not a significant predictor of protective behaviors (although it showed significant correlations with it).

Nevertheless, it should be noted that in this study, empathy was measured as a context-related factor, as empathy toward people in forced isolation. In the early stage of the pandemic in Serbia, people in forced isolation included the elderly as the main vulnerable group, but also people who came to Serbia from abroad. Thus, this measure had a narrower scope compared to measures of empathy used in other research (e.g., Pfattheicher et al., 2020).

The results also confirmed the significance of fear related to the pandemic as a positive predictor of protective behaviors, which is in line with previous studies (e.g., Harper et al., 2020). It should be noted that among context-related factors, as opposed to empathy, fear had unique contribution to protective behavior. This result highlights the important role of fear as a strong underlying mechanism of compliance with protective measures. However, fear did not act as a moderator and it seems that, among those with higher selfishness, raising the adaptive fear of pandemic could not change the health-irresponsible behavior.

There are some limitations of the study. First, the sample was convenient, recruited online via social networks. It mostly comprised highly educated participants, which limited the conclusions. Second, the study was limited to the early stage of the COVID-19 pandemic. Cao et al. (2020) showed that empathy and prosocial tendencies decreased in the post-outbreak period. Thus, a different pattern could be expected in longitudinal studies. Third, in the present study, fear was assessed as a general state related to the pandemic. It was not specified whether it referred to fear for oneself or others. Some previous studies showed that fear for relatives was stronger than fear for oneself (e.g., Akdeniz et al., 2020). Therefore, we could expect a different pattern of relations if this was taken into account.

Compared to previous research in which only prosocial (e.g., Blagov, 2020) or only antisocial tendencies (e.g., Nowak et al., 2020) were examined, in this study effects of both types of tendencies were explored since these two tendencies are not simply opposite poles (e.g., Krueger et al., 2001). Results support the view of protective behaviors as forms of prosocial and unselfish behaviors. Furthermore, the antisocial and selfish strategy can decrease the chances of both personal survival and the survival of group members. Finally, one of the novelty contribution of this study is that situational empathy could be seen as the motivational mechanism that could enhance protective behaviors among those who are characterized as more selfish. These findings have practical implications for shaping public messages and they can help effectively promote protective behaviors.

## Data Availability Statement

The datasets presented in this study can be found in online repositories. The names of the repository/repositories and accession number(s) can be found below: Open Science Framework - https://osf.io/gdz42/?view_only=620512fd2ebc484ba53d9a194f58a5a5.

## Ethics Statement

The studies involving human participants were reviewed and approved by Ethical Committee of the Department of Psychology, Faculty of Philosophy, University of Novi Sad, Serbia, which is the Second Instance Commission of the Ethical Committee of the Serbian Psychological Society (No. 202003221959_nytc). The patients/participants provided their written informed consent to participate in this study.

## Author Contributions

BD and BB contributed to the conception and design of the study, collected the data, and performed the analyses. BD wrote the draft of the manuscript. BB provided substantial feedback on the manuscript. All authors contributed to the article and approved the submitted version.

## Conflict of Interest

The authors declare that the research was conducted in the absence of any commercial or financial relationships that could be construed as a potential conflict of interest.

## References

[B1] Abdelrahman M. (2020). Personality traits, risk perception, and protective behaviors of arab residents of Qatar during the COVID-19 pandemic. Int. J. Ment. Health Addict. 22, 1–12. 10.1007/s11469-020-00352-7 32837433 PMC7307935

[B2] Akdeniz G. Kavakci M. Gozugok M. Yalcinkaya S. Kucukay A. Sahutogullari B. (2020). A survey of attitudes, anxiety status, and protective behaviors of the university students during the COVID-19 outbreak in Turkey. Front. Psychiatry 11:695. 10.3389/fpsyt.2020.00695 32760303 PMC7373786

[B3] Aschwanden D. Strickhouser J. E. Sesker A. A. Lee J. H. Luchetti M. Stephan Y.. (2020). Psychological and behavioural responses to coronavirus disease 2019: the role of personality. Eur. J. Pers. 35, 51–66. 10.1002/per.2281 32836766 PMC7361622

[B4] Ashton M. C. Lee K. de Vries R. E. (2014). The HEXACO honesty-humility, agreeableness, and emotionality factors: a review of research and theory. Pers. Soc. Psychol. Rev. 18, 139–152. 10.1177/1088868314523838 24577101

[B5] Blagov P. S. (2020). Adaptive and dark personality in the COVID-19 pandemic: predicting health-behavior endorsement and the appeal of public-health messages. Soc. Psychol. Personal. Sci. 10.1177/1948550620936439 PMC7342937 38602980

[B6] Bogg T. Milad E. (2020). Demographic, personality, and social cognition correlates of coronavirus guideline adherence in a U.S. sample. Health Psychol. 39, 1026–1036. 10.1037/hea0000891 33252928

[B7] Böhm M. Frey N. Giannitsis E. Giannitsis E. Sliwa K. Zeiher A. M. (2020) Coronavirus disease 2019 (COVID-19) and its implications for cardiovascular care: expert document from the German Cardiac Society and the World Heart Federation. Clin. Res. Card. 109, 1446–1459. 10.1007/s00392-020-01656-3 32462267 PMC7252421

[B8] Buss D. M. (2019). Evolutionary Psychology. The New Science of the Mind. 6th Edn. New York, NY: Routledge.

[B9] Cao S. Qi Y. Huang Q. Wang Y. Han X. Liu X.. (2020). Emerging Infectious Outbreak Inhibits Pain Empathy Mediated Prosocial Willingness. Lancet [preprint]. 10.2139/ssrn.3793565

[B10] Carlo G. Hausmann A. Christiansen S. Randall B. A. (2003). Sociocognitive and behavioral correlates of a measure of prosocial tendencies for adolescents. J. Early Adolesc. 23, 107–134. 10.1177/0272431602239132

[B11] Carlo G. Randall B. A. (2002). The development of a measure of prosocial behaviors for late adolescents. J. Youth Adolesc. 31, 31–44. 10.1023/A:1014033032440

[B12] Coelho C. M. Suttiwan P. Arato N. Zsido A. N. (2020). On the nature of fear and anxiety triggered by COVID-19. Front. Psychol. 11:581314. 10.3389/fpsyg.2020.581314 33240172 PMC7680724

[B13] Diebels K. J. Leary M. R. Chon D. (2018). Individual differences in selfishness as a major dimension of personality: a reinterpretation of the sixth personality factor. Rev. Gen. Psychol. 22, 367–376. 10.1037/gpr0000155

[B14] Dinić B. M. Bodroža B. (2020). “My precious… toilet paper”: Stockpiling during the COVID-19 pandemic is related to selfishness, but not to fear. Prim. Psihol. 13, 489–504. 10.19090/pp.20.4.489-504

[B15] Dinić B. M. Wertag A. Sokolovska V. Tomašević A. (2020). Centrality and redundancy of the Dark Tetrad traits. Pers. Individ. Dif. 155:109621. 10.1016/j.paid.2019.109621

[B16] Gilbert P. Basran J. (2019). The evolution of prosocial and antisocial competitive behavior and the emergence of prosocial and antisocial leadership styles. Front. Psychol. 10:610. 10.3389/fpsyg.2019.00610 31293464 PMC6603082

[B17] Graziano W. G. Habashi M. M. Sheese B. E. Tobin R. M. (2007). Agreeableness, empathy, and helping: a person x situation perspective. J. Pers. Soc. Psychol. 93, 583–599. 10.1037/0022-3514.93.4.583 17892333

[B18] Harper C. A. Satchell L. P. Fido D. Latzman R. D. (2020). Functional fear predicts public health compliance in the COVID-19 pandemic. Int. J. Ment. Health Addict. 10.1007/s11469-020-00281-5 32346359 PMC7185265

[B19] Hayes A. F. Little T. D. (2018). Introduction to Mediation, Moderation, and Conditional Process Analysis: A Regression–Based Approach. 2nd Edn. New York, NY: The Guilford Press.

[B20] Jordan J. Yoeli E. Rand D. G. (2020). Don't get it or don't spread it? Comparing self-interested versus prosocial motivations for COVID-19 prevention behaviors. PsyArxiv [preprint]. 10.31234/osf.io/yuq7x PMC8511002 34642341

[B21] Jørgensen F. J. Bor A. Lindholt M. F. Petersen M. (2020). Lockdown evaluations during the first Wave of the COVID-19 pandemic. PsyArxiv [preprint]. 10.31234/osf.io/4ske2

[B22] Krueger R. F. Hicks B. M. McGue M. (2001). Altruism and antisocial behavior: independent tendencies, unique personality correlates, distinct etiologies. Psychol. Sci. 12, 397–402. 10.1111/1467-9280.00373 11554673

[B23] Lüdecke D. von dem Knesebeck O. (2020). Protective behavior in course of the COVID-19 outbreak—survey results from Germany. Front. Public Health 8:572561. 10.3389/fpubh.2020.572561 33072712 PMC7543680

[B24] Mihić L. J. Novović Z. Colović P. Smederevac S. (2014). Serbian adaptation of the positive and negative affect schedule (PANAS): its facets and second–order structure. Psihologija 47, 393–414. 10.2298/PSI1404393M

[B25] Moshagen M. Hilbig B. E. Zettler I. (2018). The dark core of personality. Psychol. Rev. 125, 656–688. 10.1037/rev0000111 29999338

[B26] Nakayachi K. Ozaki T. Shibata Y. Yokoi R. (2020). Why do Japanese people use masks against COVID-19, even though masks are unlikely to offer protection from infection? Front. Psychol. 11:1918. 10.3389/fpsyg.2020.01918 32849127 PMC7417658

[B27] Nowak B. Brzóska P. Piotrowski J. Sedikides C. Zemojtel-Piotrowska M. Jonason P. K. (2020). Adaptive and maladaptive behavior during the COVID-19 pandemic: the roles of dark triad traits, collective narcissism, and health beliefs. Pers. Individ. Dif. 167:110232. 10.1016/j.paid.2020.110232 32834282 PMC7363424

[B28] Paciello M. Fida R. Cerniglia L. Tramontano C. Cole E. (2013). High cost helping scenario: the role of empathy, prosocial reasoning and moral disengagement on helping behavior. Pers. Individ. Dif. 55, 3–7. 10.1016/j.paid.2012.11.004

[B29] Pfattheicher S. Nockur L. Böhm R. Sassenrath C. Petersen M. B. (2020). The emotional path to action: empathy promotes physical distancing and wearing of face masks during the COVID-19 pandemic. Psychol. Sci. 31, 1363–1373. 10.1177/0956797620964422 32993455

[B30] Raine A. Uh S. (2019). The selfishness questionnaire: egocentric, adaptive, and pathological forms of selfishness. J. Pers. Assess. 101, 503–514. 10.1080/00223891.2018.1455692 29671625

[B31] Rodrigues J. Ulrich N. Mussel P. Carlo G. Hewig J. (2017). Measuring prosocial tendencies in Germany: sources of validity and reliablity of the revised prosocial tendency measure. Front. Psychol. 8:2119. 10.3389/fpsyg.2017.02119 29270144 PMC5723663

[B32] Seitz B. M. Aktipis A. Buss D. M. Alcock J. Bloom P. Gelfand M.. (2020). The pandemic exposes human nature: 10 evolutionary insights. PNAS 117, 27767–27776. 10.1073/pnas.2009787117 33093198 PMC7668083

[B33] Sheth K. Wright G. C. (2020). The usual suspects: do risk tolerance, altruism, and health predict the response to COVID-19? Rev. Econ. Househ. 18, 1041–1052. 10.1007/s11150-020-09515-w 33132793 PMC7585485

[B34] Shook N. J. Sevi B. Lee J. Oosterhoff B. Fitzgerald H. N. (2020). Disease avoidance in the time of COVID-19: the behavioral immune system is associated with concern and preventative health behaviors. PLoS ONE 15:e0238015. 10.1371/journal.pone.0238015 32817714 PMC7446877

[B35] Thielmann I. Spadaro G. Balliet D. (2020). Personality and prosocial behavior: a theoretical framework and meta-analysis. Psych. Bull. 146, 30–90. 10.1037/bul0000217 31841013

[B36] Triberti S. Durosini I. Pravettoni G. (2021). Social distancing is the right thing to do: Dark Triad behavioral correlates in the COVID-19 quarantine. Pers. Individ. Dif. 170:110453. 10.1016/j.paid.2020.110453

[B37] Vieira J. Pierzchajlo S. Jangard S. Marsh A. Olsson A. (2020). Perceived threat and acute anxiety predict increased everyday altruism during the COVID-19 pandemic. PsyArxiv [preprint]. 10.31234/osf.io/n3t5c

[B38] Watson D. Clark L. A. Tellegen A. (1988). Development and validation of brief measures of positive and negative affect: the PANAS scales. J. Pers. Soc. Psychol. 54, 1063–1070. 10.1037/0022-3514.54.6.1063 3397865

[B39] World Health Organization (2020). Coronavirus Disease (COVID-19) Advice for the Public. Available online at: https://www.who.int/emergencies/diseases/novel-coronavirus-2019/advice-for-public (accessed December 29, 2020).

[B40] Zajenkowski M. Jonason P. K. Leniarska M. Koyakiewicz Z. (2020). Who complies with the restrictions to reduce the spread of COVID-19?: personality and perceptions of the COVID-19 situation. Pers. Individ. Dif. 166:110199. 10.1016/j.paid.2020.110199 32565591 PMC7296320

[B41] Zettler I. Schild C. Lilleholt L. Kroencke L. Utesch T. Moshagen M.. (2020). The role of personality in COVID-19 related perceptions, evaluations, and behaviors: findings across five samples, nine traits, and 17 criteria. PsyArxiv [Preprint]. 10.31234/osf.io/pkm2a

