# Supplementary material for: COVID-19 Protective Behaviors Are Forms of Prosocial and Unselfish Behaviors
Source: Front Psychol. 2021 Apr 9;12:647710. doi: 10.3389/fpsyg.2021.647710 (PMC8062771; doi:10.3389/fpsyg.2021.647710)
Supplement: Supplementary file 1 [file Table_1.DOCX]

Supplementary Material

# Tables

# Table A

*Full correlation matrix between protective behaviors, context-related factors, and personality factors*

|  | 1 | 2 | 3 | 4 | 5 | 6 | 7 | 8 | 9 | 10 | 11 | 12 | 13 |
| --- | --- | --- | --- | --- | --- | --- | --- | --- | --- | --- | --- | --- | --- |
| 1 Protective behaviors | 1 |  |  |  |  |  |  |  |  |  |  |  |  |
| Context-related factors |  |  |  |  |  |  |  |  |  |  |  |  |  |
| 2 Empathy towards people in forced isolation | .20 | 1 |  |  |  |  |  |  |  |  |  |  |  |
| 3 Fear related to pandemic | .19 | .19 | 1 |  |  |  |  |  |  |  |  |  |  |
| Personality factors |  |  |  |  |  |  |  |  |  |  |  |  |  |
| 4 Selfishness total | -.21 | -.38 | .12 | 1 |  |  |  |  |  |  |  |  |  |
| 5 Adaptive selfishness | -.19 | -.27 | .13 | .91 | 1 |  |  |  |  |  |  |  |  |
| 6 Egocentric selfishness | -.19 | -.40 | .08 | .85 | .65 | 1 |  |  |  |  |  |  |  |
| 7 Pathological selfishness | -.17 | -.35 | .10 | .89 | .73 | .64 | 1 |  |  |  |  |  |  |
| 8 Dire prosociality | .09 | .16 | -.01 | -.10 | -.04 | -.17 | -.06 | 1 |  |  |  |  |  |
| 9 Public prosociality | -.02 | -.10 | .14 | .29 | .23 | .24 | .32 | .05 | 1 |  |  |  |  |
| 10 Anonymous prosociality | .20 | .22 | .00 | -.23 | -.18 | -.27 | -.16 | .21 | -.05 | 1 |  |  |  |
| 11 Compliant prosociality | .15 | .19 | -.01 | -.22 | -.16 | -.25 | -.19 | .43 | -.04 | .28 | 1 |  |  |
| 12 Emotional prosociality | .11 | .21 | .13 | .01 | .06 | -.06 | .01 | .58 | .10 | .16 | .39 | 1 |  |
| 13 Altruism | .08 | .11 | -.13 | -.46 | -.42 | -.36 | -.43 | -.10 | -.47 | .06 | .04 | -.17 | 1 |

*Note*. All correlations ≥ .13 are significant at *p* < .001.
